# KIF15 facilitates gastric cancer via enhancing proliferation, inhibiting apoptosis, and predict poor prognosis

**DOI:** 10.1186/s12935-020-01199-7

**Published:** 2020-04-15

**Authors:** Lixian Ding, Bin Li, Xiaotong Yu, Zhongsheng Li, Xinglong Li, Shuwei Dang, Qiang Lv, Jiufeng Wei, Haixia Sun, Hongsheng Chen, Ming Liu, Guodong Li

**Affiliations:** 1 Department of General Surgery, The Fourth Affiliated Hospital of Harbin Medical University, No. 37 Yiyuan Street, Nangang District, Harbin, 150001 Heilongjiang China; 2 Bio-Bank of Department of General Surgery, The Fourth Affiliated Hospital of Harbin Medical University, No. 37 Yiyuan Street, Nangang District, Harbin, 150001 Heilongjiang China; 3 Department of Clinical Laboratory, The Fourth Affiliated Hospital of Harbin Medical University, Harbin, 150001 Heilongjiang China

**Keywords:** KIF15, Gastric cancer, Apoptosis, Proliferation, Prognosis

## Abstract

**Background:**

Kinesin superfamily proteins (KIFs) can transport membranous organelles and protein complexes in an ATP-dependent manner. Kinesin family member 15 (KIF15) is overexpressed in various cancers. However, the function of KIF15 in gastric cancer (GC) is still unclear.

**Methods:**

GC patients’ data from The Cancer Genome Atlas (TCGA) were analyzed by bioinformatics methods. The expression of KIF15 was examined in GC and paracarcinoma tissues from 41 patients to verify the analysis results. The relationship between KIF15 expression and clinical characteristics were also observed by bioinformatics methods. Kaplan–Meier survival analysis of 122 GC patients in our hospital was performed to explore the relationship between KIF15 expression levels and GC patients’ prognosis. KIF15 was downregulated in GC cell lines AGS and SGC-7901 by transfecting a lentivirus-mediated shRNA plasmid targeting KIF15. In vitro, GC cell proliferation and apoptosis were detected by MTT assay, colony formation assay, and Annexin V-APC staining. In vivo, xenograft experiments were used to verify the in vitro results. Furthermore, Human Apoptosis Antibody Array kit was used to screen possible targets of KIF15 in GC cell lines.

**Results:**

The bioinformatics results showed that KIF15 expression levels were higher in GC tissues than in normal tissues. IHC showed same results. High expression of KIF15 was statistical correlated with high age and early histologic stage. Kaplan–Meier curves indicated that high KIF15 expression predict poor prognosis in patients with GC. MTT assay and colony formation assay showed that KIF15 promote GC cell proliferation. Annexin V-APC staining found that KIF15 can inhibit GC cell apoptosis. Xenograft experiments reveal that downregulating KIF15 can inhibit GC tumor growth and promote GC apoptosis. Through detection of 43 anti-apoptotic proteins by the Human Apoptosis Antibody Array kit, it was confirmed that knocking down KIF15 can reduce seven anti-apoptotic proteins expression.

**Conclusions:**

Taken together, our study revealed a critical role for KIF15 to inhibit GC cell apoptosis and promote GC cell proliferation. KIF15 may decrease anti-apoptotic proteins expression by regulating apoptosis pathways. High expression of KIF15 predicts a poor prognosis in patients with GC. KIF15 might be a novel prognostic biomarker and a therapeutic target for GC.

## Background

Cancer is the second leading cause of death worldwide, among which gastric cancer (GC) is one of the most common malignancies of the digestive system, ranking second in cancer-related mortality [1]. The main causes of GC death are rapid proliferation, invasion, metastasis, and anti-cancer drug resistance [2, 3]. The prognosis of patients can be significantly improved by the combination of radiotherapy, neoadjuvant therapy, and other treatment measures [4, 5]. However, symptoms of early GC are inconspicuous. An important factor in the gastric cancer-related mortality is the usual “advanced stage at the diagnosis” [6]. Therefore, the occurrence and development mechanisms of GC remain to be investigated.

Abnormal cellular processes such as proliferation and apoptosis in cancer cells are significant causes of cancer progression. Recent studies have found that kinesin motor proteins could facilitate cancer progression via promoting cell proliferation [7]. Increasing evidence demonstrate that kinesin proteins play carcinogenesis role in the initiation and progression of human cancers [8]. Kinesin superfamily proteins (KIFs) belong to kinesin motor proteins, which can support intracellular transport such as RNA, intracellular vesicles, and other materials [9, 10]. Up to date, over 600 members of the kinesin superfamily have been identified in addition to more than 45 members of mammals [11]. Increasing studies [8, 12–16] have shown that KIFs is related to the initiation and progression of breast cancer, lung cancer, and pancreatic cancer. Moreover, KIFs may be therapeutic targets for non-small cell lung cancer (NSCLC) [17].

Kinesin family member 15 (KIF15), a member of KIFs, belongs to the kinesin-12 subfamily, and is the only motor protein which is structurally similarly to kinesin-5 [18, 19]. KIF15 localization and motility can be regulated by the C-terminus of TPX2 and microtubule dynamics. The regulation of KIF15 makes centrosome separate during bipolar spindle assembly [20, 21]. Recently, KIF15 was reported to be overexpressed in several types of human diseases such as glioma and breast cancer [22, 23], and may facilitate these diseases. Moreover, KIF15 has been reported to promote development of digestive tumors such as pancreatic cancer [16]. However, the effects of KIF15 in GC has not been reported by consulting literatures online.

In this study, we attempted to investigate the function of KIF15 in GC through in vitro and in vivo experiments. Data from The Cancer Genome Atlas (TCGA) database were downloaded to analyze the relationship between KIF15 expression level and clinicopathologic features of GC patients. MTT and colony formation assays were performed to detected cell proliferation. Annexin V-APC staining was used to assess the apoptosis rate change in GC cells. GC xenograft mouse models were established to verify the results of in vitro experiments. Mechanistically, Human Apoptosis Antibody Array kit including 43 proteins was used to screening potential apoptotic targets of GC in GC. In summary, KIF15 serves a promoting role in GC and might be an early diagnosis marker.

## Methods

### Human samples

Clinicopathological data of GC patients were downloaded from TCGA database. Informed consent was obtained from all 122 GC patients who underwent survival follow-up in our hospital. All GC tissues enrolled in this study were taken from the Bio-Bank of Department of General Surgery in the Fourth Affiliated Hospital of Harbin Medical University. The experiment has been approved by the Medical Ethics Committee of the Fourth Affiliated Hospital of Harbin Medical University. Ethical approval number is YXLLSC-2018-01. All of the patients underwent gastrectomy, of which 41 GC tissues with paired paracarcinoma tissues from patients were obtained for the immunohistochemical assay. The specimens were pathologically diagnosed as GC. All patients have signed informed consent. All experiments were performed with mycoplasma-free cells.

### Cell culture

GC cell lines (AGS, SGC-7901, BGC-823 and MGC-803) and normal human gastric epithelial cell line GES-1 were bought from Shanghai Institutes for Biological Sciences, China. These cells were cultured with Dulbecco’s Modified Eagle medium (DMEM) medium supplemented with 10% fetal bovine serum (FPS), 100 U/ml of penicillin, and 100 μg/ml of streptomycin (HyClone).

### Immunohistochemistry (IHC)

For IHC assay, 5-mm sections from formalin-fixed-paraffin embedded tissue were transferred to polylysine-coated slides and incubated with primary antibodies against KIF15 (1:50, fine test, Wuhan, Hubei, China). Subsequently, the GTVision Two-step Visualization System (Genetech, Shanghai, China) was used to visualize the immunostaining, and hematoxylin was used to counter-stain. Finally, the sections were examined under light microscopy and evaluated by the Immuno-Reactive-Score (IRS), which was calculated by multiplying staining proportion score (PS) and staining intensity score (IS). PS was defined as 0 (0%), 1 (1–25%), 2 (26–50%), 3 (51–75%), and 4 (76–100%). IS was classified as 0 (negative staining), 1 (weak), 2 (moderate) and 3 (strong).

### Gene knockdown

BR-V-108 carrier was used in this experiment. Green fluorescent protein (GFP) was consulted in the lentivirus. After the design software was evaluated and determined, the following sequences were selected as interference targets: GCTGAAGTGAAGAGGCTCAAA. The lentivirus-containing short hairpin RNA (shRNA) targeting KIF15 was purchased from Biosci Res (Shanghai, China). The shRNAs were transfected into the GC cell lines using Lipofectamine 3000 (Invitrogen) according to the manufacturer s instructions. Each group infected the cells with 40 μl of lentivirus at a concentration of 1 × 10^8^ TU/ml. At 48 h post-transfection, the cells were selected with puromycin (2 μg/ml) for 2 weeks to construct stable cell lines. The transfection efficiency was verified by light microscopy, fluorescence microscope, qRT-PCR and WB.

### RNA extraction and qRT-PCR

To determine the knockdown efficiency, RNA samples were isolated from the collected cell lines. RNA extraction was performed using Trizol reagent (Invitrogen, Waltham, MA, USA) following the protocols. Then the RNA sample was reversely transcribed to cDNA using M-MLV RT (Promega Corporation, Madison, USA). Real-time PCR was carried out using the SYBR Premix Ex Taq reaction system, and cycling conditions were set according to the manufacturer’s instructions. GAPDH was employed as an internal control for different samples. qRT-PCR primers were all obtained from Sangon Biotech (Shanghai, China). Knockdown levels were calculated using the 2^(−ΔΔCt)^ method. The primers for gene application were listed as follows: KIF15-F5-CTCTCACAGTTGAATGTCCTTG and KIF15-R5-CTCCTTGTCAGCAGAATGAAG; and GAPDH-F5-TGACTTCAACAGCGACACCCA and GAPDH-R5-CACCCTGTTGCTGTAGCCAAA.

### Western blot (WB)

To detect the expression level of KIF15 protein, cells were lysed and total proteins were denatured after quantification. The same amount of protein was loaded on sodium dodecyl sulfate (SDS) polyacrylamide gels for electrophoresis, and then electrophoretically transferred onto polyvinylidene difluoride (PVDF) membranes. Non-fat milk powder with a concentration of 5% was used to block the non-specific sites. The blots were then incubated with various primary antibodies at 4 °C overnight. Fluorescent-based anti-rabbit IgG secondary antibody (Beyotime, Shanghai, China) was used to detect the captured primary antibodies on the membranes. Protein bands were visualized by ECL reagent (Bio-Rad, Hercules, Canada) and imaged with the ChemiDoc XRS System (Bio-Rad, Hercules, CA, USA). Finally, Image J Software was used to quantify the proteins.

### Cell proliferation and colony formation assay

Cells were digested with trypsin and cultured into suspension and counted. The cells were placed into 96-well plates, and cultured for 5 days. To determine the cell proliferation, 20 μl of 5 mg/ml 3-(4,5-dimethylthiazol-2-yl)-2,5-diphenyltetrazolium bromide (MTT, Sigma) solution was added into each well. After another 4 h incubation, supernatants were discarded, and 100 μl dimethyl sulfoxide (DMSO, Sigma) was supplemented to dissolve the formazan crystals. Finally, the absorbance was determined on a microplate reader at a wavelength of 490 nm (Bio-Rad).

For colony formation assays, cells were seeded in six-well plates at a density of 2 ml/well with each experimental group being plated in three wells. The cells were incubated over a period of 8 days. The cell colonies were fixed with 1 ml paraformaldehyde each well (Sinopharm Group, Beijing, China) for 30 min followed by Giemsa staining (500 μl/well; Shanghai Dingguo, Shanghai, China) for 20 min. Finally, the colonies were photographed and counted by fluorescence microscopy (Olympus, Japan). Each experiment was performed in triplicate.

### Apoptosis assay and tunnel staining

sh-KIF15 cell and sh-Ctrl cell cultures were trypsinized in the logarithmic growth phase for 1 h. Both adherent and floating cells were collected and washed three times with ice-cold PBS (Phosphate Buffered Saline). Subsequently, Annexin V-APC staining was used to assess the apoptosis according to the manufacturer’s instructions. Flow Cytometer (eBioscience, California, USA) was used to analyzed the GFP and Annexin V-APC of the stained cells within 15 min. GFP showed green fluorescence, and Annexin V-APC showed red fluorescence. Each experiment was performed in triplicate.

Tumor sections from GC xenograft mouse model were taken and tunel staining was performed for 1 h according to the instructions. The ratio of red light to blue light was observed by fluorescence microscope, and the apoptosis rate was calculated.

### Establishment of GC xenograft mouse model and in vivo imaging assay

Cells at concentration 1 × 10^7^ cells/ml with or without KIF15 knockdown were injected subcutaneously into the back next to the right forelimb of the nude mouse, and permitted to grow until palpable. Each mouse was injected with 200 μl. Both sh-Ctrl and sh-KIF15 group were consisted of six mice. The tumor growth was observed on the fifth day. Then tumors were measured every 3 days with a vernier caliper until the eighteenth day. Tumor volume was calculated according to the following formula. Tumor volume (mm^3^) = π/6 × L×W × W (W, width at the widest point; L, perpendicular width). Before the mice were sacrificed at the eighteenth day, vivisection imager Lumina LT (Perkin Elmer, Massachusetts, USA) was used for in vivo imaging, and fluorescence quantitative analysis was performed. All subcutaneous tumor tissues were harvested, weighted, fixed with 4% paraformaldehyde, and embedded, resected, stained with hematoxylin-eosin (HE) as well as Ki-67 to evaluate proliferation.

### Protein array analysis

shRNA was used to knockdown KIF15 mRNA expression in GC cell line SGC- 7901. Analysis of proteins by Human Apoptosis Antibody Array kit (ab134001, Abcam, Cambridge, UK) was conducted following the manufacturer’s instructions. The intensity score of each array spot was measured by the ImageJ software program. Apoptotic proteins expression levels were detected by comparing the sh-KIF15 group with the sh-Ctrl group.

### Statistical analysis

All the experiments were conducted two or three times. The statistical analyses in the current study were carried out using the software of GraphPad Prism version 8.0.1 (GraphPad Inc, San Diego, CA, USA). The Student’s *t* test was used to analyse difference between two groups. High and low age groups were divided by the median age of all patients. ANOVA was used to compare the statistical differences in more than three groups. Kaplan–Meier survival analysis and the log-rank test were used for patient survival analysis. The correlation between KIF15 and seven apoptotic genes was calculated using Spearman’s correlation. Values of P less than 0.05 were considered to indicate a significant statistically difference.

## Results

### KIF15 expression level is higher in human GC tissues

**Fig. 1:**
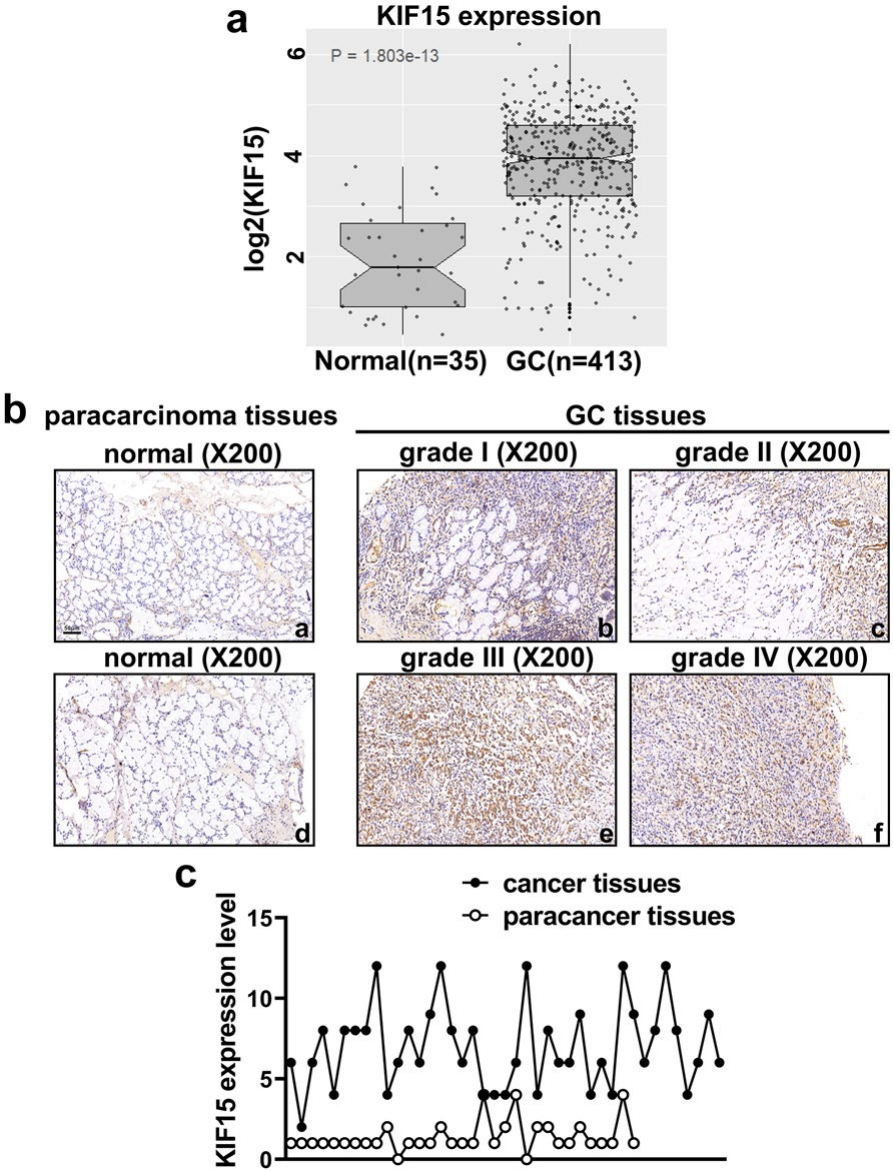
KIF15 expression is up-regulated in human GC tissues. **a** RNA sequencing data were obtained from TCGA. Statistical differences in expression between human GC tissues and paracarcinoma tissues were analyzed (P < 0.001). **b** KIF15 expression level was detected by IHC and the results were quantified according to the IHC scoring criteria. KIF15 was upregulated in all grades of GC tissues. **c** Tissue microarray analysis showed that KIF15 expression level was higher in GC tissues compared with normal tissues (P < 0.001)

The gene expression profiling data from TCGA database was examined to preliminarily investigate the role of KIF15 in GC. Human GC tissues and normal tissues were analyzed. As shown in Fig. 1a, KIF15 mRNA expression level was significantly higher in GC tissues than that in the normal tissues (P < 0.001). IHC assay further verified that KIF15 protein expression levels in human GC tissues (n = 41) were significantly higher than the matched paracarcinoma tissues (P < 0.001, Fig. 1b, c). Taken together, KIF15 expression is up-regulated in human GC tissues.

### KIF15 knockdown inhibits proliferation and promotes apoptosis in GC cells

**Fig. 2:**
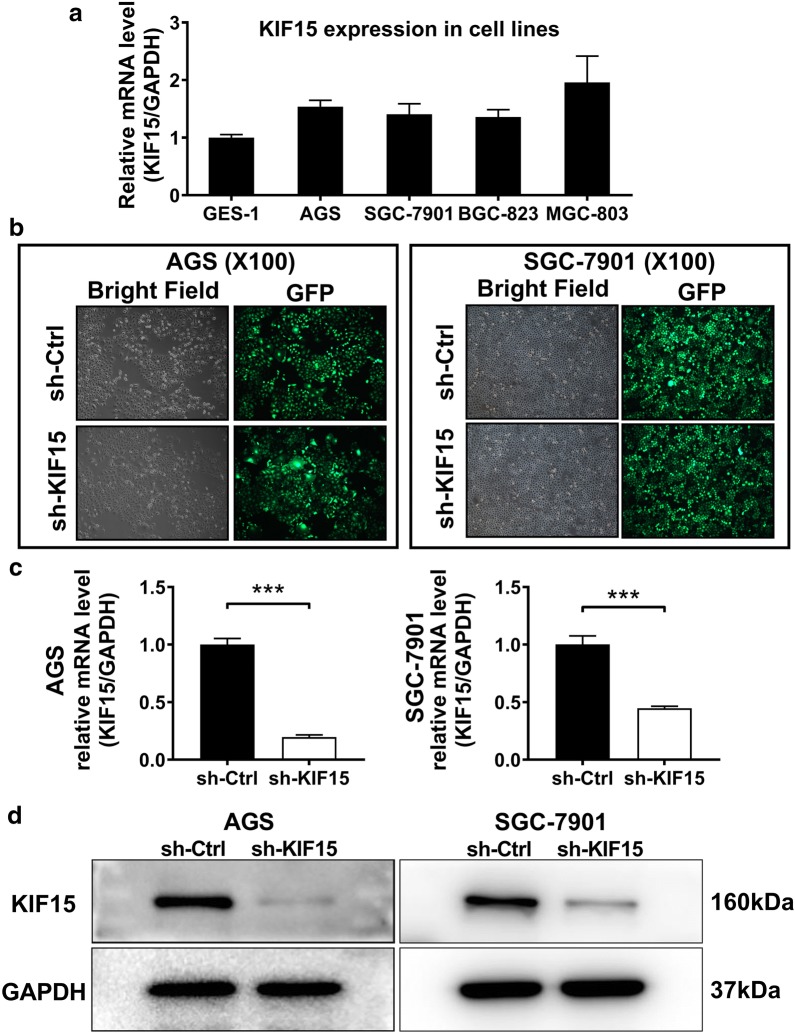
Comparison of KIF15 expression levels in different GC cell lines and knockdown efficiency of KIF15 in two GC cell lines. **a** The mRNA expression level of KIF15 was higher in GC cell lines compared with normal gastric cell line. **b** The results of bright and dark microscopy fields showed that the transfection efficiency of sh-KIF15 in two GC cell lines was significant. **c** RNA was extracted from shKIF15-AGS, shKIF15-SGC-7901 cells, and corresponding control cells. qRT-PCR proved that KIF15 expression level was significantly decreased in sh-KIF15 group in AGS and SGC-7901 cells compared with sh-Ctrl group (P < 0.001). **d** WB results also proved that the protein expression level of KIF15 was downregulated in sh-KIF15 group compared with sh-Ctrl group in GC cell lines AGS and SGC-7901. GAPDH was used as an internal control

After the comparison of KIF15 expression levels in four GC cell lines by qRT-PCR, AGS and SGC-7901 with higher and more stable expression level of KIF15 were selected for the following experiments (Fig. 2a). KIF15 were knocked down by shRNA targeting KIF15 to clarify the function of KIF15 in human GC cells. Cell status and transfection efficiency after transfection were observed under the bright and dark microscope fields respectively (Fig. 2b). The results of qRT-PCR showed that the mRNA content of KIF15 was significantly down-regulated after sh-KIF15 transfection (P < 0.001, Fig. 2c). In addition, the successful knockdown of KIF15 in AGS and SGC-7901 cells was also verified by WB (Fig. 2d).

**Fig. 3:**
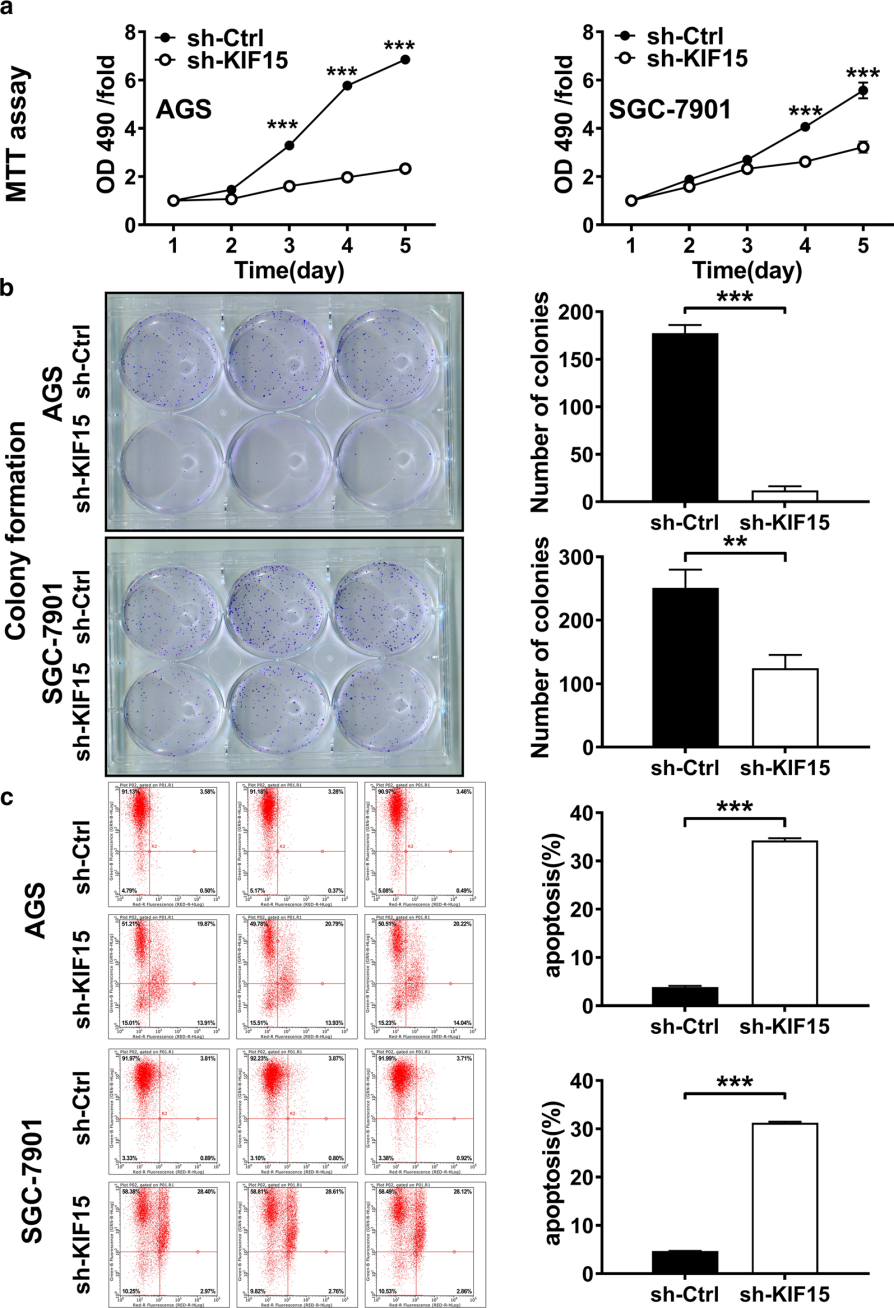
Knocking down KIF15 can inhibit cell proliferation and promote apoptosis in vitro. **a** MTT assay showed that cell growth was significantly inhibited when KIF15 was knocked down in both AGS and SGC-7901 cell lines (P < 0.001). **b** Colony formation assay showed that cell colony formation ability was inhibited after KIF15 knocking down (AGS, P < 0.001; SGC-7901, P < 0.01). **c** Flow cytometry for cell apoptosis indicated that AGS and SGC-7901 cells with KIF15 silencing exhibited significantly increased apoptotic rate compared with the control group (P < 0.001)

MTT assay was conducted to investigate the function of KIF15 in GC cell growth, and the results came out that KIF15 knockdown markedly restrained the proliferation in AGS and SGC-7901 cell lines (P < 0.001, Fig. 3a). The cell colony formation experiment showed that the clonal formation ability of the AGS and SGC-7901 cell lines decreased significantly in sh-KIF15 group than in sh-Ctrl group (AGS, P < 0.001; SGC-7901, P < 0.01, Fig. 3b). These results suggested that KIF15 can promote the proliferation of GC cells.

Annexin V-APC staining and flow cytometry were then performed with AGS and SGC-7901 cells to observe the effects of KIF15 knockdown on cell apoptosis. Flow cytometry measurement showed that the percentage of apoptotic GC cells in the both cell lines was remarkably elevated in sh-KIF15 group than sh-Ctrl group (P < 0.001, Fig. 3c). These findings indicate that KIF15 could inhibit the progression of cell apoptosis significantly in GC cells.

### KIF15 knockdown inhibits GC tumor growth in xenograft mouse model

**Fig. 4:**
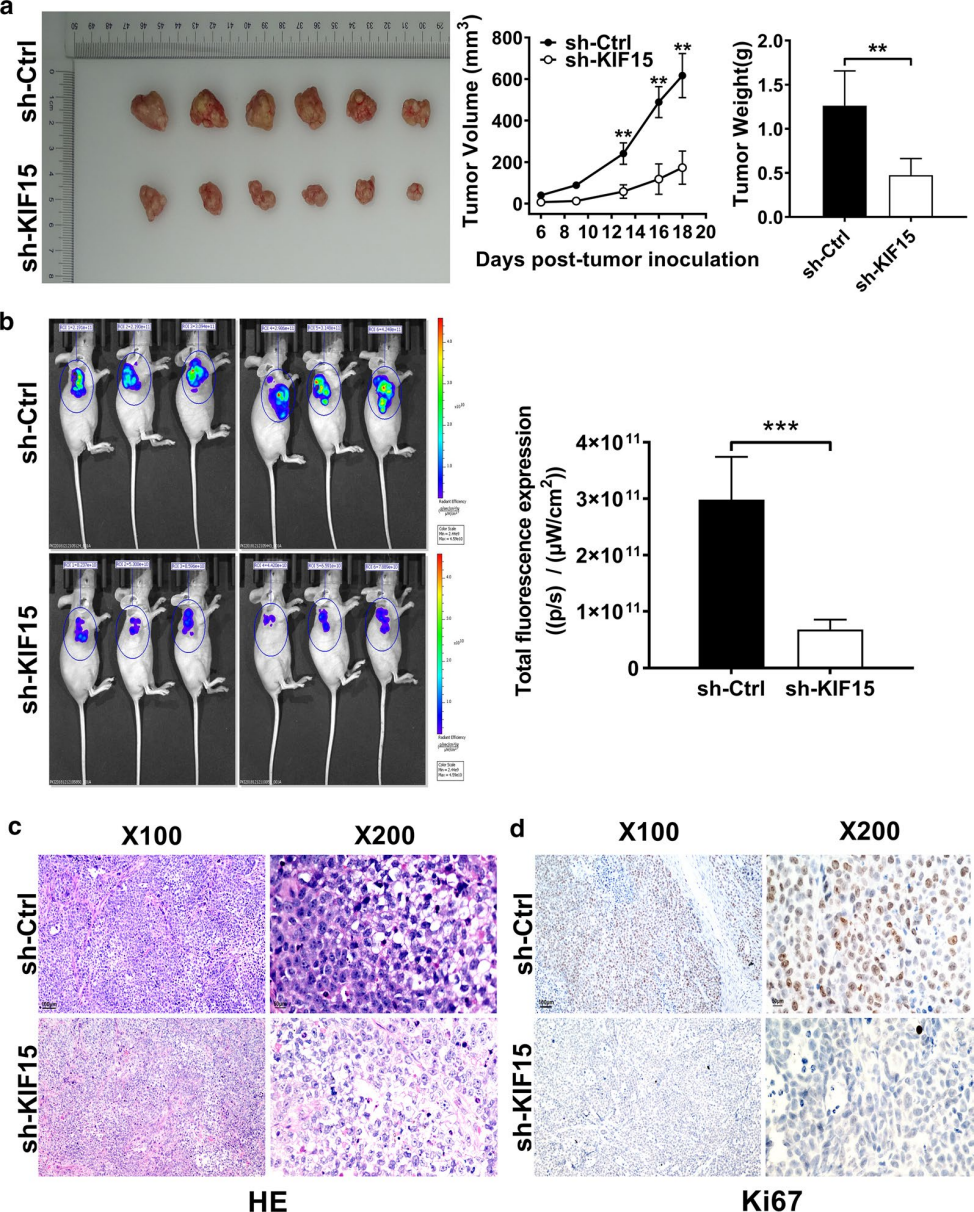
Knocking down KIF15 can suppress GC tissues growth and facilitate apoptosis in vivo. **a** Mice tumor volume curves and final tumor weight for subcutaneous xenografts showed that GC tissues growth were suppressed in sh-KIF15 group compared with sh-Ctrl group (P < 0.01). **b** Fluorescence expression in vivo was detected in mice, and was significantly decreased in the sh-KIF15 group (P < 0.001). **c** Through HE staining, GC tissues were confirmed in both groups under the microscope of 100 times and 200 times. **d** Under the microscope of 100 times and 200 times, Ki-67 staining was reduced in sh-KIF15 group compared with sh-Ctrl group

To further explore whether KIF15 plays a role in GC development in vivo, xenograft model was established based on nude mice through subcutaneous injection of SGC-7901 cells treated with sh-KIF15 or sh-Ctrl. Tumor volume measurements in mice demonstrate that the sh-KIF15 group showed a slower increase in tumor volume compared with the sh-Ctrl group (P < 0.01, Fig. 4a). Results of tumor weight measurement and in vivo bioluminescence imaging showed that down-regulation expression of KIF15 could inhibit the development of GC in mice (P < 0.01, Fig. 4b).

After the mice were sacrificed, the tumors were removed for HE staining and Ki-67 staining. HE staining verified that tissues in both groups were GC tissues (Fig. 4c). In addition, Ki-67 staining was reduced in the KIF15 knockdown group compared with the control group (Fig. 4d). The above data showed that KIF15 knockdown could inhibit the proliferation of GC cells in vivo.

### KIF15 knockdown promotes cell apoptosis in xenograft mouse model

**Fig. 5:**
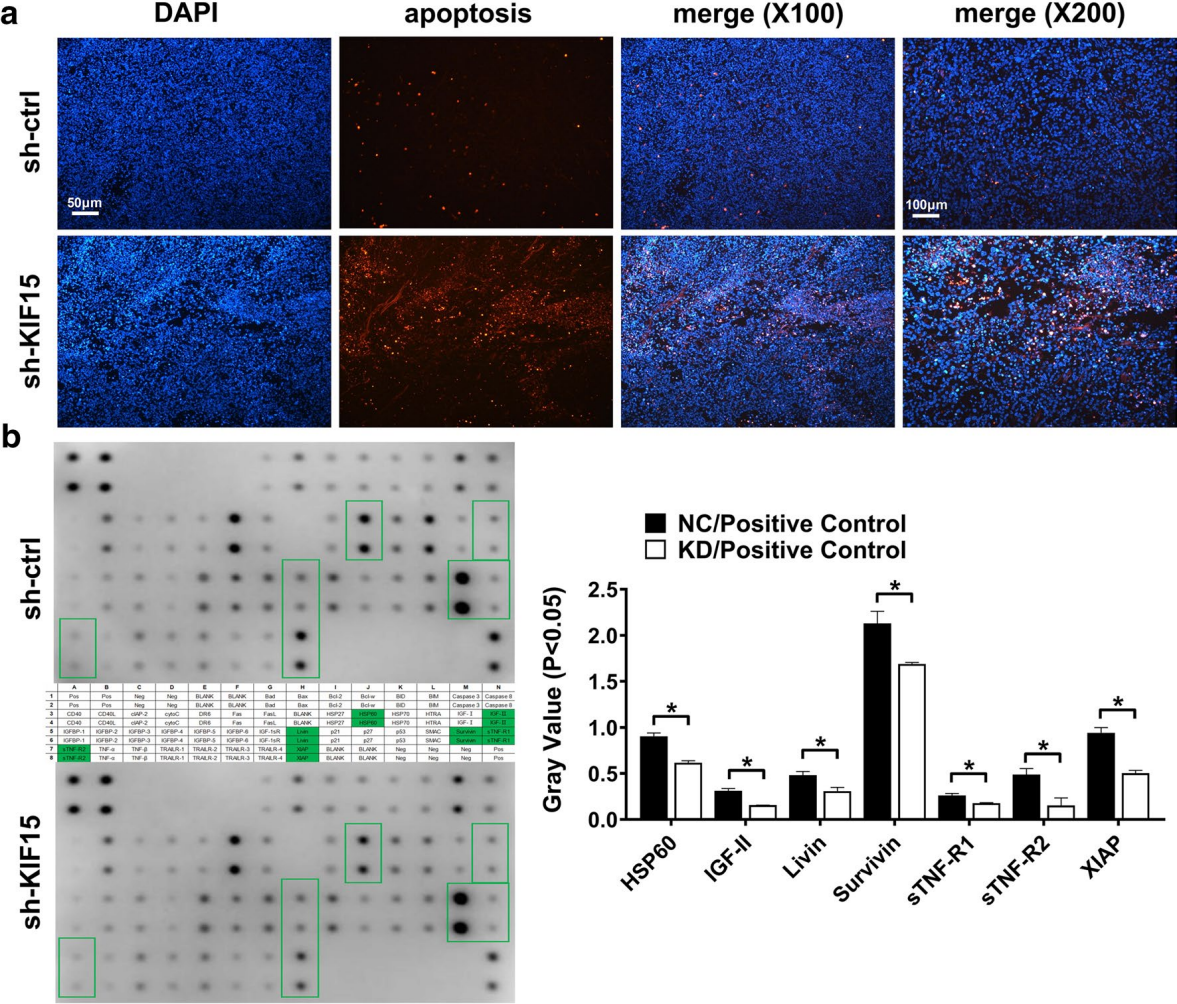
Knockdown of KIF15 promotes GC apoptosis in vivo, and decreases expression of seven apoptotic proteins in vitro. **a** Tunnel assay showed that GC cell apoptosis were markedly up-regulated when KIF15 was knocked down in vivo. **b** The protein antibody kit showed that the expression levels of seven apoptotic proteins decreased significantly in sh-KIF15 group compared with sh-Ctrl group (P < 0.05)

In order to further detect the effects of KIF15 on GC cell apoptosis in vivo, biopsy and tunnel staining in nude mouse transplantation model were performed. As shown in Fig. 5a, the apoptosis rate of tunnel cells in KIF15 knockdown group was significantly higher than that in the control group (P < 0.05). This result proves that KIF15 can inhibit apoptosis of GC in xenograft mouse model.

### KIF15 inhibits apoptosis by regulating apoptotic proteins expression

To further identify the underlying mechanisms of KIF15 induced regulation of cell apoptosis, a Human Apoptosis Antibody Array kit (ab134001), which includes 43 human apoptosis related proteins, was used. As shown in Fig. 5b, the sTNF-R2 protein expression level was reduced by 68.8%, and IGF-II was reduced by 50.19% in KIF15 knockdown group (P < 0.05). HSP60, Livin, Survivin, sTNF-R1, and X-linked inhibitor of apoptosis protein (XIAP), which are well-known as apoptotic factors, were also reduced by KIF15 knockdown treatment (HSP60, 31.85%; Livin, 35.86%; Survivin, 20.68%; sTNF-R1, 32.44%; and XIAP, 46.5%)(P < 0.05, Fig. 5b). These results suggest that KIF15 could inhibit apoptosis of GC cells through regulating the expression of the above apoptotic proteins. The corresponding diagram of the Human Apoptosis Antibody Array protein microarray kit is shown in Additional file 1: Table S1.

### Clinicopathological significance of KIF15 expression in human GC tissues

**Fig. 6:**
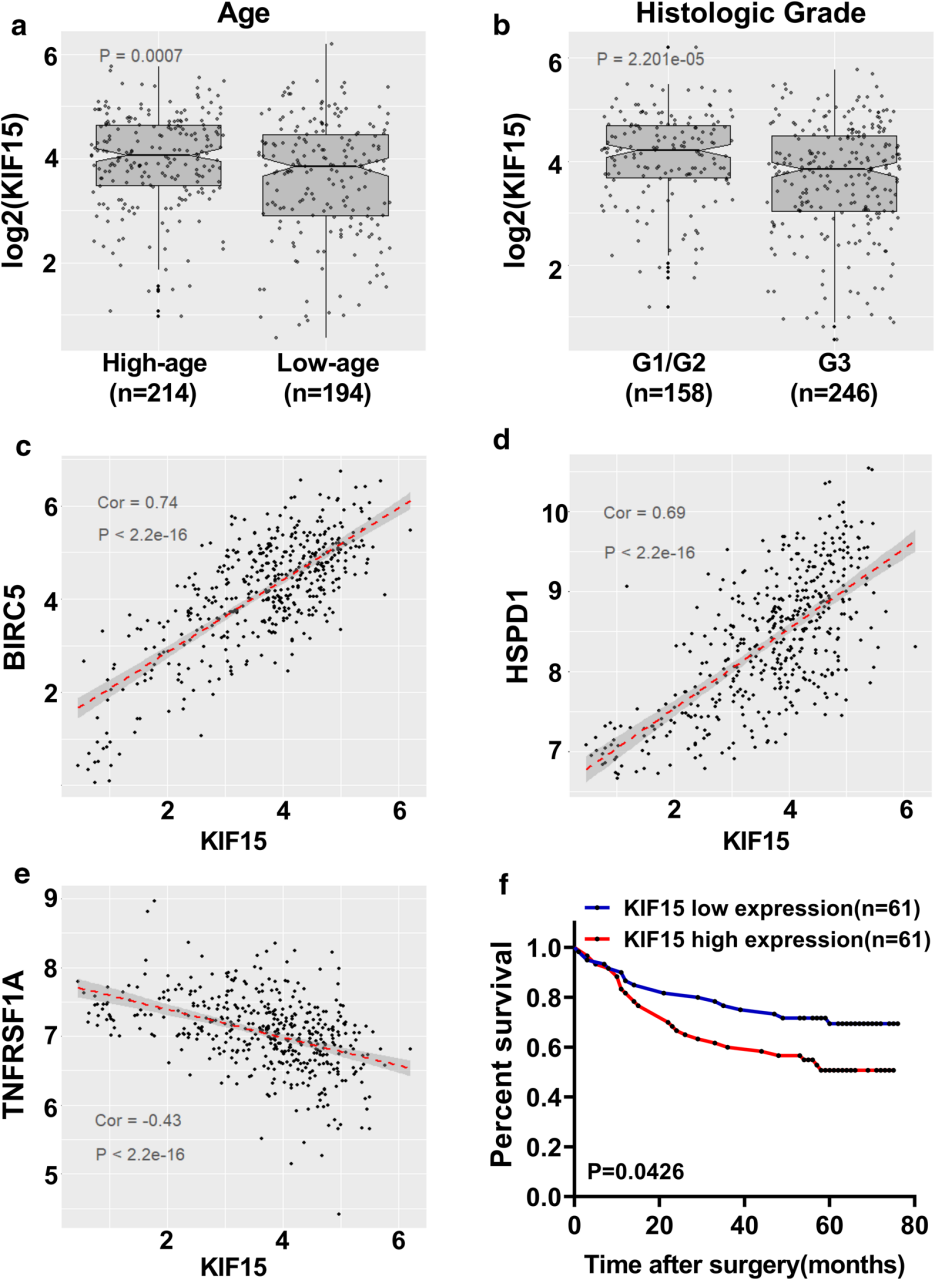
High expression levels of KIF15 is related with several clinicopathological characteristics, and predicts poor prognosis of GC patients. **a** KIF15 expression level was higher in elder (> 67) GC patients (P < 0.001). **b** KIF15 expression level was markedly increased in low GC tumor grade (G1/G2) than in high GC tumor grade (G3) (P < 0.001). **c**, **d** Regression analysis of correlation of BIRC5, HSPD1 gene expression levels and KIF15 expression level in GC tissues (Spearman r = 0.74 and 0.69 respectively, P < 0.001). **e** TNFRSF1A expression was inversely correlated with KIF15 expression in TCGA database (Spearman r = − 0.43, P < 0.001). **f** Kaplan–Meier curves of overall survival was analyzed according to KIF15 expression levels in 122 patients with GC. The result showed that higher expression level of KIF15 represents poor prognosis (P < 0.01)

Next, the association between KIF15 mRNA expression level and the clinicopathological characteristics of patients with GC in TCGA database were analyzed. As shown in Fig. 6, KIF15 mRNA expression level was statistical correlated with age (P < 0.001, Fig. 6a) and histologic stage (P < 0.001, Fig. 6b), but not with gender, race, TNM classification, and pathologic stage (Additional file 2: Figure S1A–D). KIF15 mRNA expression level was higher in elder patients (older than 67) and early histologic stage (G1/G2). Relationships between the expression of KIF15 mRNA level and clinicopathologic features of GC patients are summarized in Table 1.

**Table 1:** Relationships between the expression of KIF15 and clinicopathologic features

	KIF15 expression		P value
	Low	High	
Age			
≥ 67	97	117	0.04686
< 67	108	86	
Gender			
Male	134	133	0.9469
Female	72	74	
Race			
Asian	38	49	0.09948
White	141	117	
Pathology grade			
I	24	24	0.5214
II	60	60	
III	83	83	
IV	23	23	
T classification			
T1	10	12	0.9448
T2	46	42	
T3	89	90	
T4	57	58	
Histologic grade			
G1/G2	64	94	0.005184
G3	137	109	

High and low expression were distinguished by the median of KIF15 expression. The 67 years old is the median age of all samples

### KIF15 expression levels is correlated with several apoptotic gene expression levels in GC

The corresponding coding genes of the above seven proteins were checked on NCBI (BIRC5 codes Survivin, HSPD1 codes HSP60, TNFRSF1A codes sTNF-R1, XIAP codes XIAP, TNFRSF1B codes sTNF-R2, IGF2 codes IGF-2, BIRC7 codes Livin). Next, the RNA sequence expression data of GC patients in the TCGA database were extracted, and the correlation between KIF15 and the above 7 genes were analyzed. As shown in Fig. 6, KIF15 is significantly correlated with BIRC5, HSPD1 and TNFRSF1A (BIRC5, Spearman r = 0.74; HSPD1, Spearman r = 0.69; TNFRSF1A, Spearman r = -0.43) (P < 0.001, Fig. 6c–e). Moreover, no strong correlation was found between KIF15 and the corresponding genes of the other four proteins (Additional file 3: Figure S2A–D). These results further predicted that KIF15 could regulate GC cell apoptosis by interacting with Survivin, HSP60, or sTNF-R1 proteins.

### Correlation between KIF15 expression level and overall survival in GC patients

Given that KIF15 expression level was correlated with age and histologic stage, we further evaluated whether KIF15 expression level was associated with clinical prognosis in patients with GC. Kaplan–Meier survival analysis revealed that the survival probability of patients with high KIF15 expression level was significantly lower than that with low KIF15 expression level (P < 0.05, Fig. 6f). These results suggest that high KIF15 expression level represent poor prognosis to those patients with GC.

## Discussion

Previous studies have proposed that KIFs play important roles in different diseases even various of cancers. It can lead to congenital thrombocytopenia, breast cancer, oral cancer and bladder cancer [8, 23–26]. Recently, KIF15 has been reported to be highly expressed in gastrointestinal (GI) track cancers such as pancreatic cancer and hepatocellular cancer [16, 27]. Furthermore, abnormal high expression of KIF15 could predict a poor prognosis in patients with lung carcinoma [15]. However, the function of KIF15 in GI track disease remains to be explored. And the mechanisms of KIF15 on apoptosis in GI track cancers have not been reported as well. Here, our research proves that abnormal high expression of KIF15 is a common phenomenon in patients with GC by consulting data in TCGA and conducting IHC. Our study demonstrated that KIF15 facilitate GC via promoting cell proliferation and inhibiting cell apoptosis. Particularly, our results showed that KIF15 knockdown could lead to significantly downregulation of several apoptotic proteins such as sTNF-R2 and HSP60. Furthermore, higher expression of KIF15 was observed in elder patients (≥ 67 years old) and early GC (G1/G2). High expression of KIF15 could predict poor prognosis in patients with GC. These results indicate that KIF15 is highly expressed in the early stage of GC, and may be used in the early diagnosis of GC.

The expression level of KIF15 was found up-regulated in GC. However, its effect on GC was still unknown. It was reported that KIF15 could promote pancreatic cancer cell proliferation as a tumor promoting factor [16]. The uncontrolled proliferation of cells is an important factor in the progression of cancer. Our results of MTT assay and colony formation found that the cell viability in sh-KIF15 group is significantly decreased, and the cell proliferation ability is also remarkably reduced. This proved that KIF15 can promote proliferation of GC cells. Spindle formation is a critical step in the cell division cycle, and KIF15 is a kinase participating in spindle formation to power mitosis [28]. Mitotic centromere-associated kinesin (MCAK) is also an important protein regulating mitosis, and it could promote GC cell proliferation by promoting mitosis [29]. Therefore, we speculate that the mechanism of KIF15 to control proliferation may be similar to MCAK, which regulates mitosis to control GC cell proliferation. In addition, whether KIF15 is affected by other factors to affect cell proliferation is also worth studying.

In addition to abnormal proliferation, changes in the cell apoptosis are also key causes of cancer progression. Cell apoptosis is programmed cell death, and it plays an important role in animal tissue homeostasis [30]. The normal survival of cells require active apoptosis inhibition, which is achieved by inhibiting the expression of pro-apoptotic factors and promoting the expression of anti-apoptotic factors [31]. Abnormal regulation of apoptosis can contribute to a variety of human diseases including cancer. Recent study has reported that knockdown of KIF15 effectively promoted the apoptosis in breast cancer cells [12]. However, the function and molecular mechanism of KIF15 in regulating GC cell apoptosis has not been demonstrated.

Apoptosis has been widely studied, in which several apoptotic protein families were generally known. These apoptotic protein families, such as tumor necrosis factor receptor (TNFR) and inhibitor of apoptosis proteins (IAPs), play important roles in apoptotis pathways. TNFR includes TNR-R1, TNF-R2, and their soluble forms. It has been confirmed that TNFR was significantly higher in GC tissues than paracarcinoma tissues [32]. sTNF-R2 is the soluble form of TNF-R2, which is known as an anti-apoptotic factor. It has been reported that high expression of sTNF-R2 is associated with ovarian cancer [33]. Whether sTNF-R2 is linked to GC is worth investigating. IAPs includes XIAP, Livin, and Survivin. IAPs has also been verified to promote GC cell apoptosis and suppressing GC cell growth [34]. XIAP can inhibit Caspase-3, Caspase-7, and Caspase-9 to inhibit apoptosis [35]. Caspase, a member of cysteinyl aspartate-directed proteases that breakdown intracellular proteins, is the central regulatory molecule of cell apoptosis [36]. Once activated, Caspase hydrolyzes downstream substrates, triggering a series of events that lead to the desired biological response [37]. In addition to Caspases, XIAP can regulate apoptosis through mitochondrial pathway [38]. It have been also reported that XIAP was a kind of NF-κB-regulated gene products, and IAPs participate in many TNFR-induced pathways [25, 39, 40]. Thus, XIAP expression may be regulated by NF-κB which is associated with TNFR, and this speculation remains to be proved in GC cancers.

Therefore, we hypothesized that KIF15 may regulate the apoptosis of GC cells through these well known apoptotic factors. The Human Apoptosis Antibody Array protein kit was used in this study to detect apoptotic proteins which may be regulated by KIF15. With the results of this kit, we can infer the apoptotic pathways possibly regulated by KIF15 in GC. In our experiments, expression levels of seven common apoptotic factors were decreased markedly after silencing KIF15. Interestingly, in addition to the superfamily members mentioned above, the expression levels of IGF-2 and HSP60 were also significantly reduced when KIF15 was knocked down. IGF-2 is another anti-apoptotic factor which can also inhibit cell apoptosis [41]. HSP60 belongs to heat shock proteins (HSPs) which are well known as tumor promoting factors [42]. Studies have shown that HSP60 could promote GC via inhibiting GC cell apoptosis [43]. All the seven proteins which were detected by The Human Apoptosis Antibody Array protein kit could suppress cell apoptosis. Therefore, we believe that KIF15 may inhibit apoptosis through regulating one or more apoptosis signaling pathways. However, the specific pathway of its regulation of apoptosis remains to be verified by further experiments. Besides the changes of the expression of these seven proteins in the kit, the expression of their corresponding genes are also correlated with the expression of KIF15 in TCGA database. From the Human Apoptosis Antibody Array protein kit results, three of the seven genes were significantly correlated with KIF15. Unlike the other two genes, TNFRSF1A is negatively correlated with KIF15 expression level. This result may be because sTNF-R1 protein is not only regulated by KIF15, but also affected by other pathway proteins. In addition, although the correlation between XIAP and KIF15 is not significant, some studies have found that survivin and XIAP can regulate GC proliferation and apoptosis [34]. These results prove that KIF15 may regulate these apoptosis proteins from another aspect.

Elevated KIF15 expression can predict a poor prognosis in patients with lung adenocarcinoma [15]. Similarly, most of GC are adenocarcinomas, thus the expression level of KIF15 may also be used to assess postoperative survival in patients with GC. The clinical data of patients with GC in the TCGA database were analyzed to explore the relationship between the expression level of KIF15 and the clinical characteristics of patients with GC. It was found that KIF15 expression level was statistically higher in patients with high age (> 67 years old) or early histologic stages (G1/G2). Combined with the common phenomenon of high expression level of KIF15 in GC, we hypothesis that high expression of KIF15 may be used to predict the progression of GC in elderly people. Histological grading suggested that KIF15 might be related to the progression of GC, and this mechanism remains to be further investigated. In addition, similar to lung cancer, the follow-up data of all 122 GC tissues in our biobank showed that patients with high expression level of KIF15 had lower 5-year survival rate than patients with low expression level of KIF15. This result showed that KIF15 could serve not only as a biomarker for early diagnosis of GC, but also as a marker for prognosis of GC patients.

Furthermore, except for proliferation and apoptosis play vital roles in GC, invasion and metastasis are important causes of poor prognosis of GC. Kinesin family member 26B (KIF26B) is a novel oncogene upregulated in metastatic GC tissues, and it could promote metastasis via VEGF pathway in GC [44]. However, no report has been published on whether KIF15 is related to the invasion and metastasis of GC so far. Moreover, it has been reported that KIF15 could assist cancer cells to develop chemotherapy resistance, thus affecting the efficiency of cancer treatment [45]. KIF15 tends to be compensatorily up-regulated when Eg5 is inhibited by anticancer drugs because of its structural and functional similarities to Eg5 in mitosis [46, 47]. Therefore, whether KIF15 plays a role in chemotherapy of GC also needs further investigation. The combined inhibitor of KIF15 and Eg5 may provide a novel strategy for figuring out chemotherapeutic resistance.

Hence, the present study verified the high expression of KIF15 in GC, and further explore the possible mechanism of promoting proliferation and inhibiting apoptosis of KIF15 in GC progression. This study also confirmed the important role of KIF15 in clinical practice, and showed KIF15 may be used as a diagnostic and prognostic factor of GC.

## Conclusions

In summary, our study finds that abnormal high expression of KIF15 is a frequent event in GC. KIF15 exerts a tumor promoting action by promoting GC cell proliferation and inhibiting apoptosis. Human Apoptosis Antibody Array kit demonstrated that anti-apoptotic mechanism of KIF15 may be achieved by inhibiting expression levels of seven apoptotic proteins. KIF15 expression level is statistically correlated with age and histologic stage in GC patients. High expression of KIF15 is related with poor overall survival possibility in patients with GC. These findings provide a novel oncogenic like factor in the progression of GC, suggesting that KIF15 may be a molecular marker with potential diagnostic and treatment value.

## Supplementary information

**Additional file 1: Table S1.** The green boxes showed seven positive anti-apoptotic proteins which were detected by the Human Apoptosis Antibody Array kit (ab134001).

**Additional file 2: Figure S1.** KIF15 expression level has no relationship with four clinicopathological features. **(A)** gender, **(B)** race, **(C)** T classification, and **(D)** pathologic stage (P > 0.05).

**Additional file 3: Figure S2.** Four positive apoptotic genes in Human Apoptosis Antibody Array kit have little correlation with KIF15. KIF15 expression level was barely association with **(A)** XIAP, **(B)** TNFRSF1B, **(C)** IGF2, and **(D)** BIRC7 (Spearman r = 0.12, − 0.02, − 0.18, 0.20, respectively).

## Data Availability

All data generated or analyzed during this study are included in this published article and its additional files.

## Abbreviations

GC: Gastric cancer
KIFs: Kinesin superfamily proteins
KIF15: Kinesin family member 15
TCGA: The Cancer Genome Atlas
shRNA: Short hairpin RNA
WB: Western blot
GFP: Green fluorescent protein
HSPs: Heat shock proteins
NSCLC: Non-small cell lung cancer
IHC: Immunohistochemistry
IRS: Immuno-Reactive-Score
TNFR: Tumor necrosis factor receptor
qRT-PCR: Quantitative real-time PCR
SDS: Sodium dodecyl sulfate
XIAP: X-linked inhibitor of apoptosis protein
IAPs: Inhibitor of apoptosis protein
PBS: Phosphate buffered saline
HE staining: Hematoxylin–eosin staining
